# Photo-assisted synthesis of coaxial-structured polypyrrole/electrochemically hydrogenated TiO_2_ nanotube arrays as a high performance supercapacitor electrode†

**DOI:** 10.1039/c7ra13166f

**Published:** 2018-04-10

**Authors:** Jiaqin Liu, Jingwei Li, Mengjia Dai, Ying Hu, Jiewu Cui, Yan Wang, Hark Hoe Tan, Yucheng Wu

**Affiliations:** Institute of Industry and Equipment Technology, Hefei University of Technology Hefei 230009 China jqliu@hfut.edu.cn; Key Laboratory of Advanced Functional Materials and Devices of Anhui Province Hefei 230009 China ycwu@hfut.edu.cn; School of Materials Science and Engineering, Hefei University of Technology Hefei 230009 China; Department of Electronic Materials Engineering, Research School of Physics and Engineering, The Australian National University Canberra ACT 2601 Australia

## Abstract

An organic–inorganic coaxial-structured hybrid of PPy/EH-TNTAs electrode with outstanding supercapacitive performance was developed by incorporating electroactive polypyrrole (PPy) into a highly-conductive TiO_2_ substrate, namely, electrochemically hydrogenated TiO_2_ nanotube arrays (EH-TNTAs) through a photo-assisted potentiodynamic electrodeposition route. The as-fabricated PPy/EH-TNTAs hybrid electrode achieves a specific capacitance of up to 614.7 F g^−1^ at 1.0 A g^−1^ with 87.4% of the initial capacitance remaining after 5000 cycles at 10 A g^−1^, outperforming other fabricated PPy-TNTAs hybrid electrodes. The photoelectrodeposited and electrodeposited hybrid samples as well as the EH-TNTAs-based and air–TNTAs-based hybrid samples were fully compared from electropolymerization process, morphology, structural feature and electrochemical perspectives. The results indicate that the synergy of remarkably improved conductivity and electrochemical properties of the TiO_2_ substrate induced by intentionally introduced Ti^3+^ (O-vacancies) as well as the homogenous and integrated deposition of PPy triggered by light illumination enabled the outstanding supercapacitive performance of the PPy/EH-TNTAs hybrid electrode. A symmetric supercapacitor device was assembled using the PPy/EH-TNTAs hybrid as both a positive and negative electrode, respectively. It displays a high energy density of 17.7 W h kg^−1^ at a power density of 1257 W kg^−1^. This organic–inorganic coaxial-structured PPy/EH-TNTAs electrode will be a competitive and promising candidate for application in future energy storage devices.

## Abstract 2

An organic–inorganic coaxial-structured hybrid of PPy/EH-TNTAs electrode was developed and applied for high performance supercapacitors.

## Graphical abstract

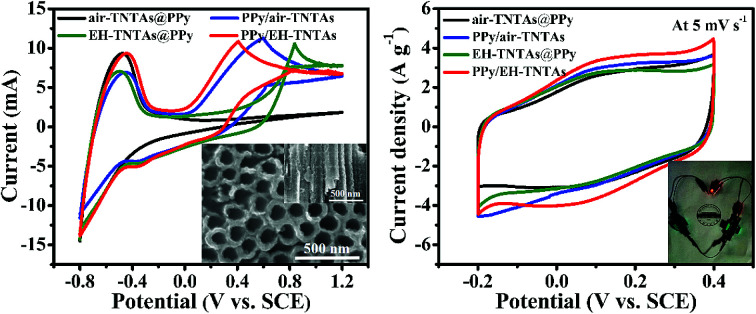

## Introduction

With an ever-growing demand in electric energy storage for electric vehicles and portable electronic devices, supercapacitors (SCs) have attracted tremendous research interest because of their high power capability, long cycle life, and fast charge–discharge rates. Generally, SCs can be classified into two categories based on their energy storage mechanism, the electrical double layer capacitors (EDLCs) and pseudo-capacitors (PCs). EDLCs physically store charge *via* reversible ion adsorption at the electrode/electrolyte interface, while PCs chemically store charge *via* fast reversible surface redox reaction. In comparison with EDLCs, whose capacitances are restricted by insufficient charge accumulation in the electrical double layer, PCs could achieve substantially higher specific capacitance and energy density through fast redox reaction, and thereby, have great promise to meet the requirements for future energy storage devices.^1–3^ Electrode material is the key component that determines the capacity and cost of SCs. Typically, carbonaceous materials are normally used for EDLCs due to their high surface area and electrical conductivity,^4,5^ while conducting polymers (CPs)^6–8^ and mental compounds^9–12^ are the two main categories of pseudo-capacitive materials for PCs. Among them, CPs such as polypyrrole (PPy), polyaniline (PANI), and polythiophene (PTh) have been extensively studied as an attractive class of electrode materials for SCs because of their high capacitance and conductivity, low cost, and fast doping/dedoping rate. However, the relatively poor cycle stability of CPs-based electrodes hinders their wide application. Hybridization of organic CPs with inorganic matrix as well as nanostructuring one or both components of hybrid have proven to be effective for reinforcing the cycle stability and maximizing the capacitances of CPs.^13,14^

Recently, well-organized frameworks of inorganic semiconductors with well-defined morphologies such as anodic TiO_2_ nanotube arrays (TNTAs) have shown great promises for various electrochemical applications.^15,16^ Moreover, TNTAs may serve as a favorable matrix for nano-structured electroactive materials to form hybrid systems owing to their high-surface-area, chemical stability, direct electron transport pathways and simple fabrication. Nonetheless, the intrinsically poor electrical conductivity of TNTAs derived from the semiconductive nature of TiO_2_ rendered them not the desirable current collectors for the application of constructing high performance SCs. Hydrogenation of TNTAs has been demonstrated to be a quite effective strategy for significantly improving the electrical conductivity and electrochemical properties of TNTAs owing to the intentionally introduced Ti^3+^ (O-vacancies) during hydrogenation process. As reported, there are many approaches for hydrogenation of TNTAs such as hydrogen thermal treatment,^17,18^ hydrogen plasma treatment,^19,20^ electrochemical hydrogenation (EH),^21,22^ and chemical hydrogenation.^23,24^ Among them, EH is a facile, low-cost and environment-friendly method, overcoming the weaknesses of other hydrogenation techniques such as expensive facility, high-energy consumption and complex process. With an external electric field, hydrogen can be driven into TiO_2_ lattice and reduces Ti^4+^ to Ti^3+^ (O-vacancies). Thus, electrochemically hydrogenated TNTAs (EH-TNTAs) with remarkably improved conductivity and electrochemical property are expected to be a high-efficiency current collector as well as an outstanding support for other high specific capacitance materials in building various high performance TNTAs-based SCs.

In order to achieve homogeneous distribution of CPs and intimate contact between CPs and semiconductor hosts, various attempts, such as chemical,^25^ thermal,^26^ or UV-assisted polymerization^27^ have been recently made for grafting organic CPs onto inorganic semiconductor host. Particularly, electrodeposition is a powerful and versatile technique because it relies on exploitation of the intrinsic electroactivity of the monomer.^28^ The foremost advantage of this approach is that CPs could be directly electrogenerated on the surface of inorganic nanostructures, which act as a working electrode. Therefore, this technique has been applied to deposit CPs onto different semiconductor matrixes such as TiO_2_,^29^ WO_3_,^30^ or ZnO.^31^ Besides, recent studies demonstrated that employing illumination during electrodeposition process helps to overcome the insufficient electroactivity of semiconductors and achieve homogeneous distribution of CPs and intimate contact between CPs and semiconductor host.^32^

Herein, highly-conductive electrochemically hydrogenated TNTAs (EH-TNTAs) was firstly employed as the inorganic semiconductor host matrix. An organic–inorganic coaxial hybrid of PPy/EH-TNTAs electrode with outstanding supercapacitive performance was developed *via* a photo-assisted electrodeposition route by electropolymerizing pyrrole onto both outer and inner surface of EH-TiO_2_ NTs with the presence of light illumination. More importantly, the crucial role of EH treatment for TiO_2_ substrate and light illumination on the electropolymerization process, morphologies, structural features and electrochemical perspectives of the resulting PPy-TNTAs hybrid electrodes as well as the corresponding mechanism are thoroughly discussed. The obtained PPy/EH-TNTAs electrode achieves a specific capacitance of up to 614.7 F g^−1^ at 1 A g^−1^ with 87.4% of the initial capacitance remaining after 5000 cycles at 10 A g^−1^. After assembling the symmetric supercapacitor device, a high energy density of 17.7 W h kg^−1^ can be achieved at the power density of 1257 W kg^−1^. This work demonstrates that the self-supported PPy/EH-TNTAs electrode has great potential for application in next generation charge storage devices.

## Experimental section

### Preparation of TiO_2_ nanotube arrays (TNTAs)

Highly ordered and well-separated TNTAs were directly grown on a Ti foil (0.1 mm, 99.7%) using electrochemical anodization. Prior to anodization, Ti foils were thoroughly cleaned by sequential ultrasonication in acetone, ethanol and deionized water. Then, anodization was performed in a two-electrode system with a Ti foil anode and a graphite foil cathode, using a solution of 0.25 M NH_4_F in ethylene glycol (EG) with 8 vol% H_2_O as electrolyte at a constant potential of 60 V for 6 h in ice bath. After anodization, the as-fabricated samples were washed with deionized water, and annealed in air at 500 °C for 2 h. The as-prepared TNTAs sample was denoted as air–TNTAs.

### Electrochemical hydrogenation of TNTAs

Electrochemical hydrogenation of TNTAs was carried out in a two-electrode setup with the as-fabricated air–TNTAs as cathode and a graphite foil as anode, using an aqueous solution of 0.1 M Na_2_SO_4_ as supporting electrolyte at a constant potential of 4 V for 20 min at ambient atmosphere. The samples were finally cleaned and dried in air at 80 °C. Similarly, the resulting TNTAs sample prepared by potentiostatic electrochemical hydrogenation was denoted as EH-TNTAs.

### Fabrication of PPy/EH-TNTAs hybrid

A photo-assisted electrodepostion approach was utilized for fabrication of PPy/EH-TNTAs hybrid. In a typical synthesis, electrodeposition of PPy onto EH-TNTAs was conducted by cyclic voltammetry (CV) on a three-electrode setup in the potential ranging from −0.8 V to 1.2 V at scan rate of 50 mV s^−1^ with the EH-TNTAs, a Pt wire, and a saturated calomel electrode (SCE) as working, counter, and reference electrodes in aqueous solution containing 0.05 M pyrrole monomer and 0.10 M H_2_SO_4_ supporting electrolyte. During the electrodepositon process, a 300 W xenon lamp was employed as the light source, and the working electrode was placed 10 cm away from the radiation source with the surface perpendicular to the light beam. Similarly, PPy/air–TNTAs hybrid was also fabricated by photo-assisted electrodepositing PPy onto air–TNTAs.

For comparison, PPy was also electrodeposited onto EH-TNTAs and air–TNTAs by CV without the presence of light illumination. Accordingly, the resulting samples were respectively denoted as EH-TNTAs@PPy and air–TNTAs@PPy.

### Symmetric supercapacitor device assembling

The symmetric supercapacitor devices were finally assembled in a two-electrode configuration using the as-fabricated PPy/EH-TNTAs hybrid as both positive and negative electrode, a cellulose membrane as separator, and 1 M H_2_SO_4_ as electrolyte. Note that the positive and negative electrodes were placed face-to-face with a separator in between and then fixed into a text fixture consisting of two glass plates.

### Characterization

Morphologies were observed using field emission scanning electron microscopy (FESEM, SU-8020, Hitachi). Structural features of as-prepared samples were identified by Raman spectroscopy (LabRAM HR Evolution, Horiba) and X-ray photoelectron spectroscopy (XPS, ESCALAB 250Xi, Thermo Scientific).

### Electrochemical measurement

The electrochemical performances of the tested samples were investigated by cyclic voltammetry (CV), galvanostatic charge–discharge (GCD) tests, and electrochemical impedance spectroscopy (EIS) studies. All electrochemical measurements were carried out on an electrochemical workstation (Autolab PGSTAT302N) in a conventional three-electrode cell using the tested sample, a Pt wire, a SCE as the working, counter, and reference electrodes, and 1 M H_2_SO_4_ aqueous solution as electrolyte. EIS measurements were performed in the frequency ranging from 100 mHz to 100 kHz at open-circuit potential with a AC-voltage amplitude of 5 mV. The energy storage performance of the assembled device was also evaluated by CV and GCD tests.

## Results and discussion

### Synthesis of organic–inorganic coaxial hybrid of PPy/EH-TNTAs

**Fig. 1:**
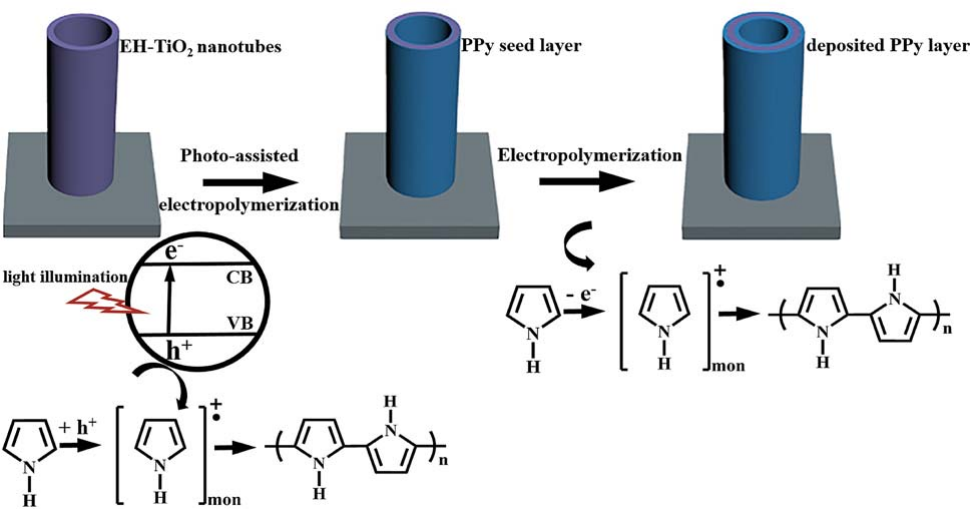
The schematic illustration for the synthesis of PPy/EH-TNTAs hybrid.

The schematic illustration for the synthesis of PPy/EH-TNTAs hybrid of is depicted in Fig. 1. As a result of light excitation, electron–hole pairs are generated in the EH-TNTAs. After charge separation, part of the photo-excited charge carriers reach the surface before they recombine. Particularly, intentionally introduced Ti^3+^ (O-vacancies) in EH-TNTAs could remarkably enhance the photoactivity and boost charge separation and transfer efficiencies (as discussed below), thereby enabling more charge carries to reach the surface and participate subsequent reactions. At the TiO_2_/electrolyte interface, holes oxidize the pyrrole monomers, accordingly generating radical cations. Then, two radical cations react with each other to form dimers that can further react with either a monomer or an oligomer radical cation. As the oligomers progressively become insoluble, thereafter, a continuous ultrathin polymer seed layer is formed on the surface of EH-TiO_2_ NTs. Meanwhile, the photo-excited electrons react with dissolved O_2_, which exists in the solution as e^−^ scavengers. Subsequently, electropolymerization will be proceeded based on the deposited seed layer.

**Fig. 2:**
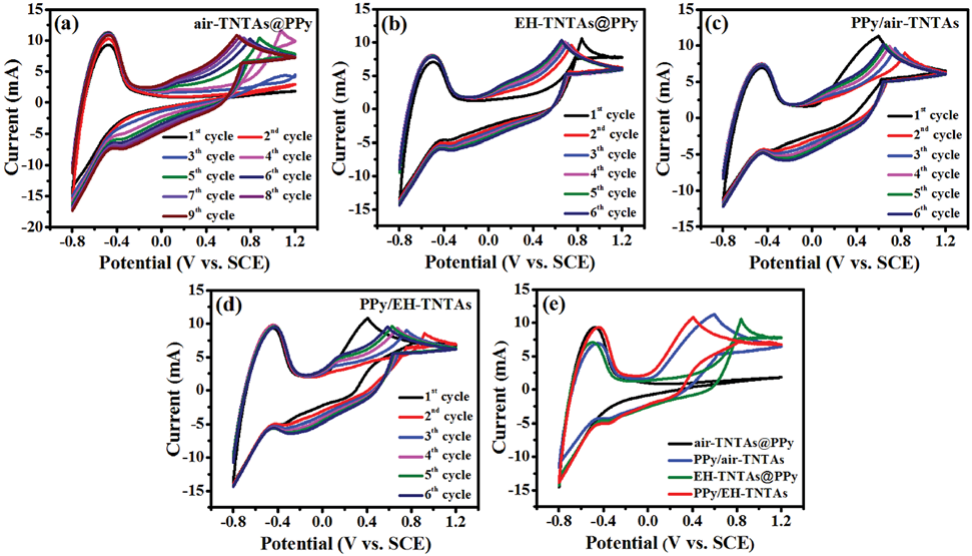
Comparison of CVs for fabrication of various PPy-TNTAs hybrids (a) air–TNTAs@PPy (dark), (b) EH-TNTAs@PPy, (c) PPy/air–TNTAs, (d) PPy/EH-TNTAs, and (e) comparison of the pattern for the 1st cycles extracted from CVs of various PPy-TNTAs hybrids.

To reveal the role of the electroactivity of different TNTAs matrix as well as the effect of light illumination on the electropolymerizaiton process, a comparison of CVs for fabrication of various PPy-TNTAs hybrids were measured, as depicted in Fig. 2. When the air–TNTAs was directly subjected to electropolymerization in the dark (Fig. 2a), there was no current response in the initial few cycles at positive voltage range, and current response occurred at the negative voltage range. This indicates that TiO_2_ is insufficiently electroactive for dark elctrodeposition of PPy on it in strong acidic environment due to the poor conductivity of wide bandgap TiO_2_. The current response at the negative voltage range is attributed to the electrochemical reduction of TNTAs, namely, electrochemical hydrogenation. As a result, from the 4th cycles, a rise of oxidation current is observed as a function of applied potential, owing to the initiation of electropolymerization. Comparatively, when the air–TNTAs was replaced by EH-TNTAs for dark electrodeposition of PPy, as can be seen in Fig. 2b, the current response occurred from the 1st cycle at the positive voltage range, revealing that EH-TNTAs is sufficiently electroactive for electropolymerization of pyrrole on it even without illumination. The dramatic enhancement in the electroactivity of EH-TNTAs mainly benefits from the greatly improved conductivity and electrochemical performance induced by the introduction of large numbers of Ti^3+^ (O-vacancies) during EH process (as discussed below). Further, anodic oxidation potential for electrodepositing PPy on EH-TNTAs (+0.84 V in the 1st cycle) is much lower than that registered during depositing PPy on air–TNTAs (+1.08 V in the 4th cycle, which was taken as the 1st active cycle), and as well, gradually shift to lower potential from +0.84 V (1st cycle) to +0.62 V (6th cycle) with increasing the cycles. Meanwhile, the oxidation current in this case is higher than that registered in electropolymerization pyrrole on air–TNTAs at identical applied potential. All this indicates that compared with air–TNTAs, EH-TNTAs is more competent to serve as a host matrix for electrodeposition of CPs.

Comparatively, electropolymerization was further carried out with both PPy/air–TNTAs and PPy/EH-TNTAs systems under illumination but otherwise identical circumstances, and corresponding CV curves were registered and depicted as Fig. 2c and d. Apparently that the CV curves for this two cases are dramatically different from that of their counterparts obtained in dark, mainly lying in the oxidation potential and pattern of 1st half-cycle. First, oxidation potentials for 1st half-cycle registered under illumination are apparently lower than that of their counterparts obtained in dark. This reveals that the oxidation potential required for photo-assisted electropolymerization of pyrrole at initial stage is much lower than that required for subsequent electropolymerization of pyrrole due to the optical shielding by the photodeposited PPy. Further, thanks to the gradual improvement of conductivity induced by the deposited PPy seed layer, the oxidation potential required for subsequent cycles gradually decreased. Moreover, photocurrent for the 1st cycle starts at *E* = 0.05 V, and develops rapidly with increasing the bias potential, and this trend is more pronounced for PPy/EH-TNTAs system due to the greatly enhanced photoactivity and electrochemical properties of EH-TNTAs matrix. To clearly illustrate the impact of light illumination and EH treatment, the pattern of 1st cycles was extracted from the CVs and depicted in Fig. 2e, and obviously that both light illumination and EH treatment for TNTAs greatly affect the electrodeposition of PPy on the TNTAs matrix.

### Morphology characterization

**Fig. 3:**
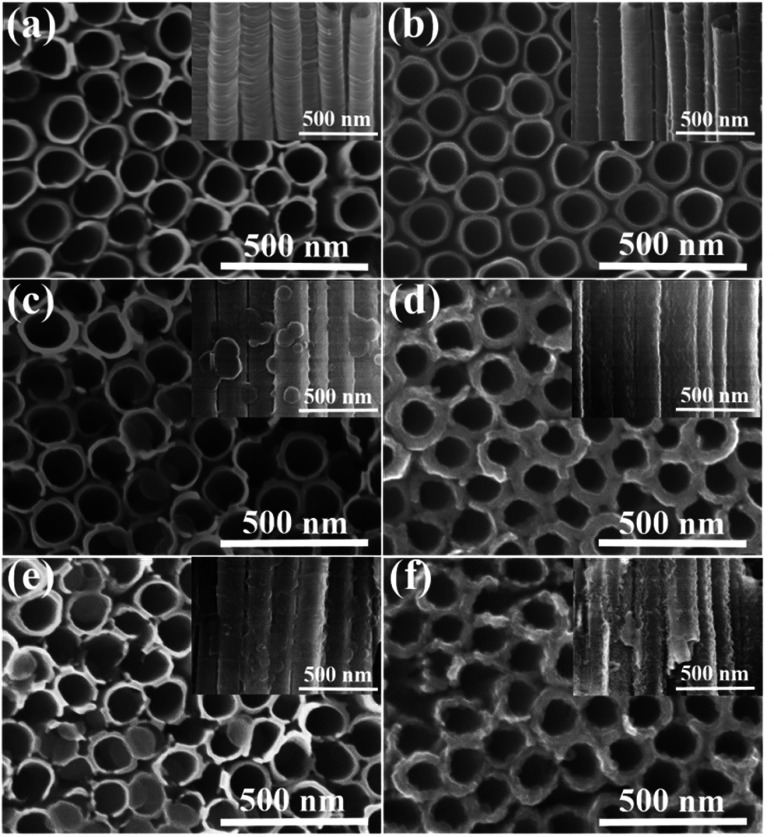
FESEM images of (a) air–TNTAs, (b) EH-TNTAs, (c) air–TNTAs@PPy, (d) PPy/air–TNTAs, (e) EH-TNTAs@PPy, (f) PPy/EH-TNTAs (insets are the corresponding side-view images).

Morphologies and microstructures of two different TNTAs supports and various PPy-TNTAs hybrids were characterized by FESEM, as shown in Fig. 3. The as-formed TiO_2_ NTs have a uniform inner diameter of ∼160 nm, a wall thickness of 20–25 nm (Fig. 3a) and a tube length of ∼10 μm (Fig. S1†). The distinct difference to the conventional TNTAs is that the TiO_2_ NTs are free-standing and well-separated from each other with intertube spacings ranging from 8 to 10 nm. Such spacings among the tubes allows for more exposed active surface as well as efficient mass transport. After EH treatment, EH-TNTAs display no distinct differences in tubular structure and surface morphology compared to air–TNTAs (Fig. 3b), which indicates the favorable electrochemical stability of TNTAs. Notably, EH-TNTAs present a dark blue colour *versus* the grey air–TNTAs, indicating a strong absorption in the visible region due to the hydrogen diffusion into TiO_2_ lattice during EH process. All microstructures of EH-TNTAs enable them to serve as a good support for the pseudocapacitive active materials to form hybrid structures. Then, various PPy-TNTAs hybrids were fabricated by electrodepositing PPy on different TNTAs matrix with six active potentiodynamic cycles under illumination or in the dark. Fig. 3d and f respectively show the representative FESEM images for the obtained hybrid samples by electrodepositing PPy on air–TNTAs and EH-TNTAs under illumination. Both the outer and inner surface of TiO_2_ NTs were fully coated with a homogeneous layer of deposited PPy. Closer inspection reveals that this PPy layer was actually composed of large amounts of tiny nanoparticles. It should be noticed that although the tube walls are completely covered by polymeric skin, the nanotubes still maintain open-end structure, which could provide favorable pathways for the infusion of electrolyte. In contrast, the morphology of hybrids obtained in the absence of illumination is strikingly different from their counterparts fabricated under illumination, as seen in Fig. 3c and e. The three most striking alterations are the following: (i) instead of forming a thin film coverage on the surface of tubular matrix, large round-like polymeric nanoflakes can be observed, randomly distributed on the inner walls of the substrate with a certain angle between them; and (ii) the polymeric nanoflakes prefer to grow along the outer walls of the substrate due to the limitation of small spacings among nanotubes; (iii) the mass of deposited polymer in the resulting hybrids obtained without illumination is obviously less than that in the case of the illuminated samples. Moreover, by precisely checking and comparing the mass of the substrate before and after deposition (Table S1†), the PPy mass in the hybrid obtained with air–TNTAs as the matrix is less than that in the counterpart with EH-TNTAs as the matrix. All these indicate that both TiO_2_ support and the light illumination remarkably affect the morphological features of these hybrids.

### Spectroscopic analysis

**Fig. 4:**
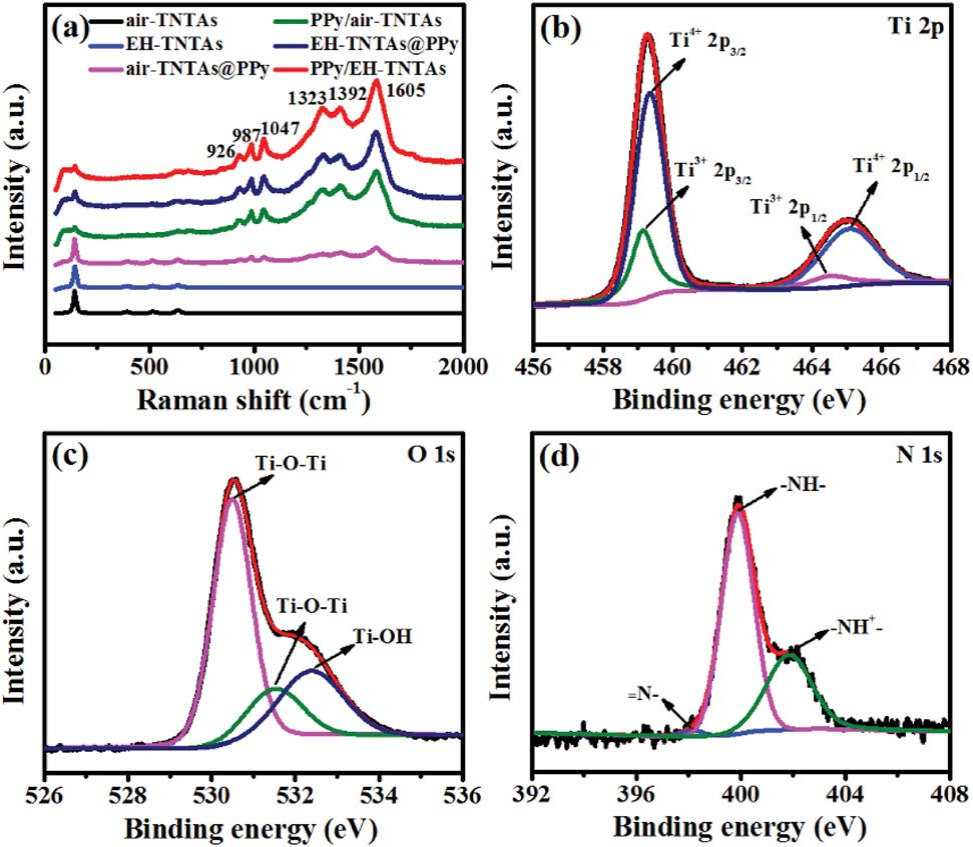
(a) Raman spectra of air–TNTAs, EH-TNTAs and various PPy-containing hybrids, XPS spectra of (b) Ti 2p, (c) O 1s and (d) N 1s of PPy/EH-TNTAs hybrid.

Raman spectroscopy was performed to confirm the identity of the electrodeposited polymers in the hybrid configurations, and also check if any permanent overoxidation of the polymer occurred during electrodeposition. Fig. 4a contains Raman spectra of two different TiO_2_ substrate and various PPy-TNTAs hybrid samples. Four Raman bands are clearly seen in the spectra of air–TNTAs and EH-TNTAs at 147, 385, 519, and 641 cm^−1^ and assigned to E_g_, B_1g_, A_1g_/B_1g_, and E_g_ active modes of anatase TiO_2_.^33^ No Raman bands related to the rutile or brookite phases are seen in the spectra of TiO_2_ substrates. Beyond the appearance of the bands originating from the TiO_2_ support, several new bands can be observed from the spectra of the PPy-containing hybrid samples. All characteristic bands can be assigned to the PPy. The band at 926 cm^−1^ corresponds to the CH out-of-plane bending, the band at 987 cm^−1^ is allotted to the ring deformation, the band at 1047 cm^−1^ is assigned to the CH in-plane bending, the bands at 1323 cm^−1^ and 1392 cm^−1^ are associated with the C–C stretching vibration peaks, and the bands at 1605 cm^−1^ is linked to the C=C backbone stretching vibration peak.^32,34^ All characteristic peaks demonstrate the formation of PPy during the electropolymerization and presence of PPy in the hybrids.

XPS studies were further conducted to identify the surface elements of PPy/EH-TNTAs hybrid and verify the deposition of PPy in the hybrid. Fig. 4b–d respectively show the Ti 2p, O 1s and N 1s XPS spectra of PPy/EH-TNTAs. Two peaks of Ti 2p (Fig. 4b) centered at 459.34 and 465.15 eV correspond to Ti 2p_3/2_ and Ti 2p_1/2_ peaks of Ti^4+^, and the other two peaks of Ti 2p located at 459.12 and 464.50 eV could be ascribed to the Ti 2p_3/2_ and Ti 2p_1/2_ peaks of Ti^3+^, proving the Ti^3+^ sites (O-vacancies) generation during hydrogenation.^35,36^ Two distinct peaks of O 1s (Fig. 4c) at 530.5 and 531.5 eV are assigned to Ti–O–Ti, and the peak at 532.4 eV could be attributed to the surface Ti–OH.^37,38^ The N 1s XPS spectrum presents three main peaks (Fig. 4d) with binding energies at 398.2, 399.9 and 401.9 eV, which respectively correspond to =N–, –NH– and –NH^+^–.^39,40^ Therefore, all XPS results again confirm the successful PPy deposition onto EH-TNTAs in the PPy/EH-TNTAs hybrid.

### Electrochemical properties

#### Electrochemical properties of TiO_2_ support

**Fig. 5:**
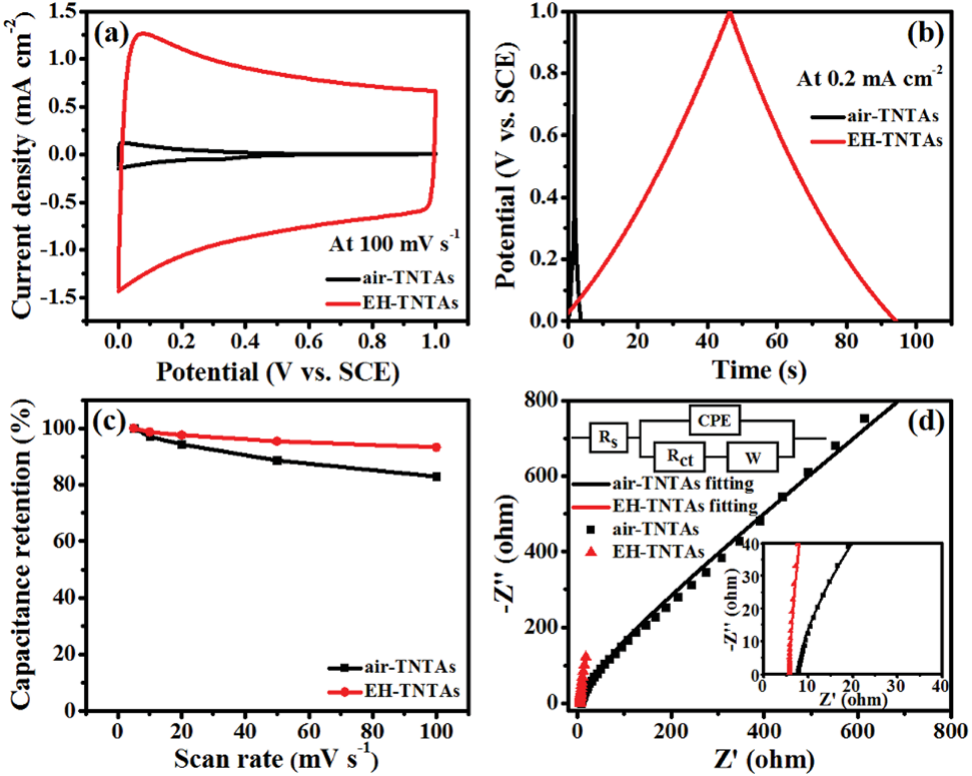
Comparison of CV curves (a), GCD curves (b), capacitance retention (c) and Nyquist plots (d) of air–TNTAs and EH-TNTAs electrodes, two insets in (d) are the magnified view of the high-frequency region and the equivalent circuit for two different TNTAs electrodes.

To investigate the effect of EH treatment on the electrochemical properties of TNTAs support, electrochemical measurements were conducted in a conventional three-electrode cell with a Pt wire counter electrode and a SCE reference electrode in 1 M H_2_SO_4_ solution. Fig. 5a compares the CV curves of air–TNTAs and EH-TNTAs electrodes collected at 100 mV s^−1^. In contrast to air–TNTAs, EH-TNTAs electrode presents an obvious pseudocapacitive characteristic, which could be ascribed to oxidation/reduction of surface hydroxyl groups.^41^ Furthermore, CV curves of EH-TNTAs collected at various scan rates exhibit quasi-rectangular shape (Fig. S2†), and the shapes of these CVs remain unaltered from 5 to 100 mV s^−1^, indicating good capacitive behavior and high-rate capability of EH-TNTAs. The areal capacitances of both air–TNTAs and EH-TNTAs electrodes as a function of scan rates were calculated and listed in Table S2.† EH-TNTAs achieves an areal capacitance of 8.58 mF cm^−2^ at 100 mV s^−1^, which is significantly higher than that of air–TNTAs (0.29 mF cm^−2^). Besides, the capacitance retentions of EH-TNTAs at various scan rates are apparently higher than that of air–TNTAs, again confirming its high-rate capability (Fig. 5c). The electrochemical performance of two different TNTAs samples was further studied by GCD measurements. Fig. 5b compares the GCD curves of two different TNTAs electrodes measured at 0.2 mA cm^−2^. Apparently, GCD curve of EH-TNTAs is symmetric and substantially prolonged over that of air–TNTAs, showing greatly improved capacitive behavior. The capacitances of air–TNTAs and EH-TNTAs derived from GCD curves measured at different current densities (Fig. S2†) were also calculated and listed in Table S3.† The results obtained from GCD tests are consistent with that of CV measurements, that is, EH-TNTAs show dramatically enhanced electrochemical performance compared to air–TNTAs. Then, EIS measurement was performed to evaluate the electrical properties of two different TNTAs electrodes. Fig. 5d presents the Nyquist plots of both air–TNTAs and EH-TNTAs electrodes. For quantitative analysis, experimental data of impedance spectra were fitted to the model depicted by the equivalent circuit (inset of Fig. 5d), which involved the elements of the solution resistance (*R*_s_), the inherent resistance of working electrode together with the charge transfer resistance (*R*_ct_), the constant phase element (CPE), and Warburg impedance (*W*). The fitting results as listed in Table S4† show that the *R*_ct_ for EH-TNTAs (16.03 Ω) is far lower than that of air–TNTAs (107.48 Ω). This dramatic decline in the resistance of EH-TNTAs could be attributed to the greatly improved conductivity and efficient charge carrier transport induced by the introduction of large numbers of Ti^3+^ (O-vacancies) during EH process. Above electrochemical analysis indicates that compared with air–TNTAs, EH-TNTAs with remarkably improved conductivity and electrochemical properties are more competent to serve as an outstanding host support for building high performance TNTAs-based SCs.

#### Electrochemical properties of PPy-TNTAs hybrids

The supercapacitive performance of various PPy-TNTAs hybrid electrodes, and as well as the PPy/Ti electrode were evaluated using CV and GCD tests, the registered CV (collected at different scan rates from 5 to 100 mV s^−1^) and GCD (collected at different current densities from 1 to 10 A g^−1^) curves were shown in Fig. S3.† As can be clearly seen, CV curves of different PPy-TNTAs electrodes exhibit rectangular-like shape and good symmetry at various scan rates, indicating the good capacitive behavior of PPy-containing electrodes. Similarly, the corresponding GCD curves at different current densities are symmetric with nearly linear slopes, also revealing the high-rate capability and good reversibility. The specific capacitances of hybrid electrodes as a function of scan rate (based on the mass of PPy) were calculated and listed in Table S5.† Among all tested hybrid electrodes, PPy/EH-TNTAs electrode shows the best supercapacitive performance. The calculated capacitance based on GCD data (Table S6†) is in good agreement with the result obtained from CVs as well. The specific capacitance of PPy/EH-TNTAs reaches up to 614.7 F g^−1^ (based on the mass of PPy), which is apparently higher than the values obtained from PPy/air–TNTAs (558.8 F g^−1^), EH-TNTAs@PPy (497.8 F g^−1^) and air–TNTAs@PPy (450.6 F g^−1^) at identical current density of 1 A g^−1^. It is worth noting that the capacitances of all PPy-TNTAs electrodes are substantially higher than the PPy/Ti electrode, fully affirming the progressive and positive role of tubular TiO_2_ substrate on the capacitive behavior of deposited PPy compared to planar Ti substrate.

**Fig. 6:**
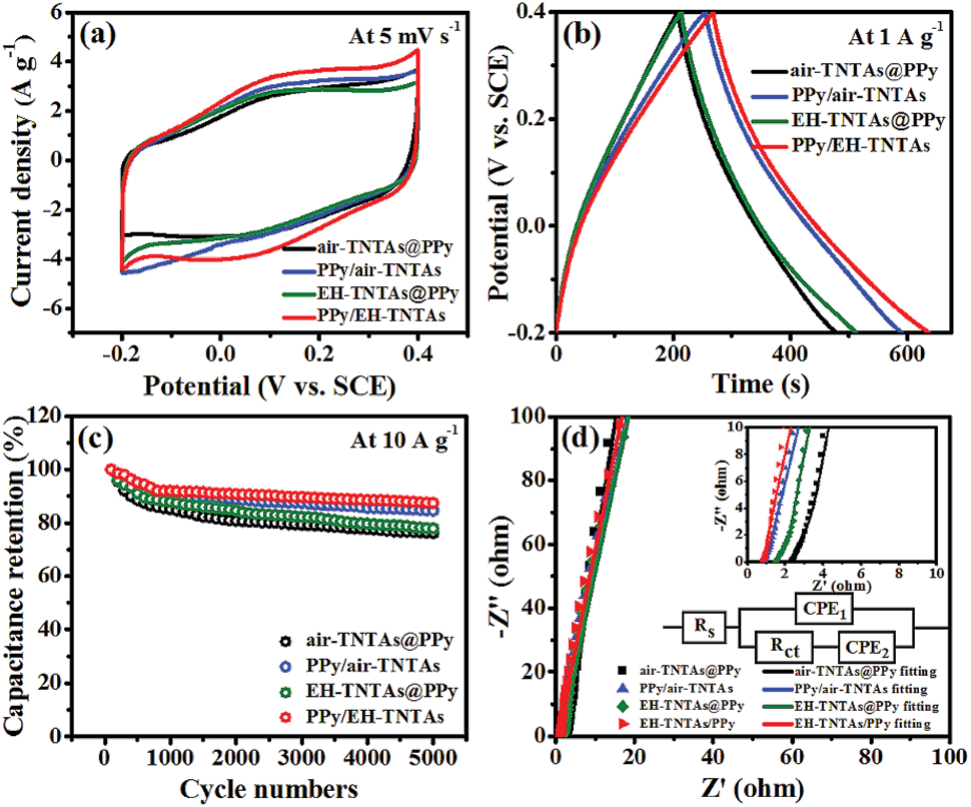
Comparison of CV curves (a), GCD curves (b), capacitance retention (c) and Nyquist plots (d) of various PPy-TNTAs electrodes, two insets in (d) are the magnified view of the high-frequency region and the equivalent circuit for all PPy-TNTAs electrodes.

How the illumination and TiO_2_ support affecting the supercapacitive performance of PPy-TNTAs hybrid were further explored. Fig. 6a and b compare the CV and GCD curves of different PPy-TNTAs hybrid electrodes collected at identical testing conditions. Apparently, the current response of the hybrids fabricated with illumination is apparently higher than that of the counterparts obtained without the absence of illumination (Fig. 6a), yet the illumination intensity does not much affect the cycling ability of PPy/EH-TNTAs hybrid, as depicted in Fig. S4.† Besides, the GCD curves of the hybrids obtained with the EH-TNTAs as the matrix is prolonged over the curves of the counterparts with the air–TNTAs as the matrix (Fig. 6b). More importantly, the PPy/EH-TNTAs electrode displays the best cyclability among all hybrid electrodes (Fig. 6c), and it retains 87.4% of its original capacitance after 5000 cycles at 10 A g^−1^, indicating the prominent stability of the deposited PPy layer during the charge–discharge process, which is favorable for practical applications. Further, EIS measurement was conducted to further investigate the electrochemical interfacial properties of the hybrid electrodes. Fig. 6d contains the typical Nyquist plots of different PPy-TNTAs hybrids and corresponding equivalent circuit, which involves the elements of bulk solution resistance (*R*_s_), the charge-transfer resistance (*R*_ct_), Warburg diffusion impedance (*W*) and the constant phase element (CPE) related to the capacitance of electrode. The fitting results summarized in Table S7† indicate that among all hybrid electrodes, PPy/EH-TNTAs delivers the lowest *R*_ct_ value (3.528 Ω), which is lower than the values of PPy/air–TNTAs (4.276 Ω), EH-TNTAs@PPy (7.590 Ω) and air–TNTAs@PPy (9.152 Ω), implying the best charge transfer ability of PPy/EH-TNTAs hybrid. This could be attributed to the greatly enhanced conductivity of EH-TNTAs support and intimate contact between the deposited PPy layer and TiO_2_ support, thereby providing easier and more efficient access for charge transfer. In addition, the *R*_ct_ values for hybrids using EH-TNTAs as the support are lower than that of the counterparts using the air–TNTAs as the support, and the *R*_ct_ values for the hybrids fabricated with illumination are lower than that of the counterparts obtained without the absence of illumination. This result well verifies above EIS result for two different TNTAs supports, and interprets the performance variation of the hybrids induced by using different TNTAs as the support, or whether illumination was utilized or not during the polymerization process.

To further explore the energy storage performance of PPy/EH-TNTAs hybrid for practical applications, a symmetric supercapacitor device was assembled in a two-electrode configuration with as-fabricated PPy/EH-TNTAs hybrid as both positive and negative electrodes. Fig. S5a and b† compare the CV and GCD curves of the as-assembled device with different working potential windows. As can be seen clearly, when the working potential window was extended to be 0–1.6 V, CV curves of the assembled device only slightly deviated from rectangular-like shape, and the responding GCD curves are still symmetric with nearly linear slope, thus the working potential window could be optimized to be 0–1.6 V. Fig. S5c† displays the CV curves of the assembled device at different scan rates ranging from 5 to 100 mV s^−1^ in the potential window of 0–1.6 V, and the calculated specific capacitances as a function of scan rates are presented in Fig. S5d,† the specific capacitance of the assembled device could reach 49.9 F g^−1^ at 5 mV s^−1^ (based on the PPy mass in both positive and negative electrode). Based on the GCD results in Fig. S5e,† energy density (*E*) and power density (*P*) are calculated, and the corresponding Ragone plot is displayed in Fig. S5f.† The largest energy density of 17.7 W h kg^−1^ can be achieved at the power density of 1257 W kg^−1^. After charging, two of this assembled symmetric supercapacitor devices connected in series are able to light up a commercial light-emitting diode (LED, red) more than 2 min (inset in Fig. S5f†), indicating the attractive potential of this kind of symmetric supercapacitor device for the smart practical applications.

## Conclusions

In summary, an organic–inorganic coaxial-structured hybrid of PPy/EH-TNTAs electrode with outstanding supercapacitive performance was fabricated by incorporating electroactive polypyrrole (PPy) into highly-conductive electrochemically hydrogenated TiO_2_ nanotube arrays (EH-TNTAs) through a photo-assisted potentiodynamic electrodeposition route. Due to the synergy of remarkably improved conductivity and electrochemical properties of TiO_2_ support induced by intentionally introduced Ti^3+^ (O-vacancies) as well as the homogenous and integrated deposition of PPy triggered by light illumination, the specific capacitance of PPy/EH-TNTAs reaches up to 614.7 F g^−1^ at 1.0 A g^−1^ with 87.4% of the initial capacitance remaining after 5000 cycles at a high current density of 10 A g^−1^, outstripping other fabricated PPy-TNTAs hybrid electrodes. After assembling the symmetric supercapacitor device, a high energy density of 17.7 W h kg^−1^ can be achieved at the power density of 1257 W kg^−1^. The PPy/EH-TNTAs hybrid may serve as one of the most attractive candidates for future high-performance energy storage electrodes.

## Conflicts of interest

There are no conflicts to declare.

## Supplementary Material

RA-008-C7RA13166F-s001
