# Supplementary material for: Photo-assisted synthesis of coaxial-structured polypyrrole/electrochemically hydrogenated TiO2 nanotube arrays as a high performance supercapacitor electrode
Source: RSC Adv. 2018 Apr 10;8(24):13393–400. doi: 10.1039/c7ra13166f (PMC9079746; doi:10.1039/c7ra13166f)
Supplement: RA-008-C7RA13166F-s001 [file RA-008-C7RA13166F-s001.pdf]

Electronic Supplementary Material (ESI) for RSC Advances.
This journal is © The Royal Society of Chemistry 2018

Supporting Information

Photo-assisted synthesis of coaxial-structured polypyrrole/electrochemically hydrogenated TiO_2 nanotube arrays as high performance supercapacitor electrode

Jiaqin Liu*[abd], Jingwei Li[a], Mengjia Dai[a], Ying Hu[ab], Jiewu Cui[bc], Yan Wang[bc], Hark Hoe Tan[d] and Yucheng Wu*[bc]

a Institute of Industry and Equipment Technology, Hefei University of Technology, Hefei 230009, China

b Key Laboratory of Advanced Functional Materials and Devices of Anhui Province, Hefei 230009, China

c School of Materials Science and Engineering, Hefei University of Technology, Hefei 230009, China

d Department of Electronic Materials Engineering, Research School of Physics and Engineering, The Australian National University, Canberra, ACT 2601, Australia

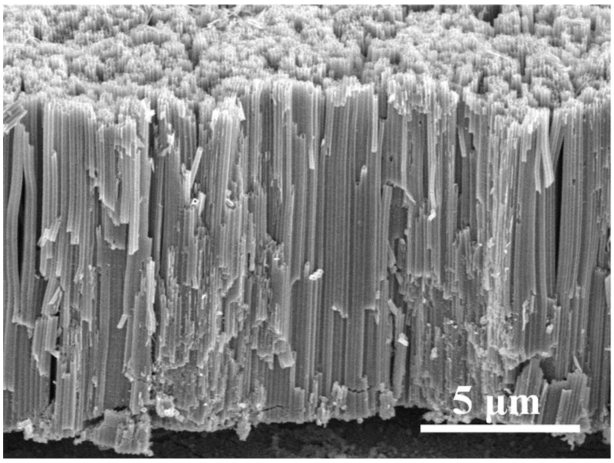

Fig. S1 Side-view FESEM image of air-TNTAs

Table. S1 Mass of electrodeposited PPy in PPy/Ti and different PPy-containing hybrids within 2 cm^2 active area

sample	mass of electrodeposited PPy (mg)
air-TNTAs@PPy	0.28
EH-TNTAs@PPy	0.31
PPy/air-TNTAs	0.35
PPy/EH-TNTAs	0.40
PPy/Ti	0.41

Capacitances calculated from CV curves:

The capacitances of electrodes were calculated from the CV curves according to the following equations:

$$C_\mathrm{s} = \frac{S}{2 \times U \times \Delta V \times S_\mathrm{W}}, \quad C_\mathrm{m} = \frac{S}{2 \times U \times \Delta V \times m}$$

where C_s (mF cm^{-2}) refers to the areal capacitances of two different TNTAs (air-TNTAs and EH-TNTAs) electrodes, S (A×V) is the integral area of CV curves, U (V s^{-1}) is the scan rates, ΔV (V) is the potential window and S_w (cm^2) is the active surface area of two different TNTAs electrodes.

C_m (F g^{-1}) refers to the specific capacitances of PPy/Ti and different PPy-containing hybrid electrodes, and m (g) is the mass of electrodeposited PPy.

Capacitances calculated from galvanostatic charge/discharge (GCD) curves:

The capacitances of electrodes measured by GCD were calculated based on the following equations:

$$C_{\mathrm{s}} = \frac{I \times \Delta t}{\Delta V \times S_{\mathrm{w}}}, \quad C_{\mathrm{m}} = \frac{I \times \Delta t}{\Delta V \times m}$$

Where C_{s} (mF cm^{-2}) refers to the areal capacitance of two different TNTAs (air-TNTAs and EH-TNTAs) electrodes, I (A) is the constant discharging current, Δt (s) is the discharging time, ΔV (V) is the potential window, and S_{w} (cm^{2}) is the active surface area of two different TNTAs (air-TNTAs and EH-TNTAs) electrodes.

C_{m} (F g^{-1}) refers to the specific capacitances of PPy/Ti and different PPy-containing hybrid electrodes, and m (g) is the mass of electrodeposited PPy.

Energy density and power density of assembled device calculated based on its GCD curves:

Energy density (E, Wh kg^{-1}) and power density (P, W kg^{-1}) of the assembled symmetric supercapacitor device were calculated based on its GCD results using the following equations：

$$E = \frac{0.5 \times C_{\mathrm{m}} \times (\Delta V)^2}{3.6}, \quad P = \frac{3600E}{\Delta t}$$

where C_{m} (F g^{-1}) refers to the specific capacitance, Δt (s) is the discharge time, ΔV (V) is the working potential window.

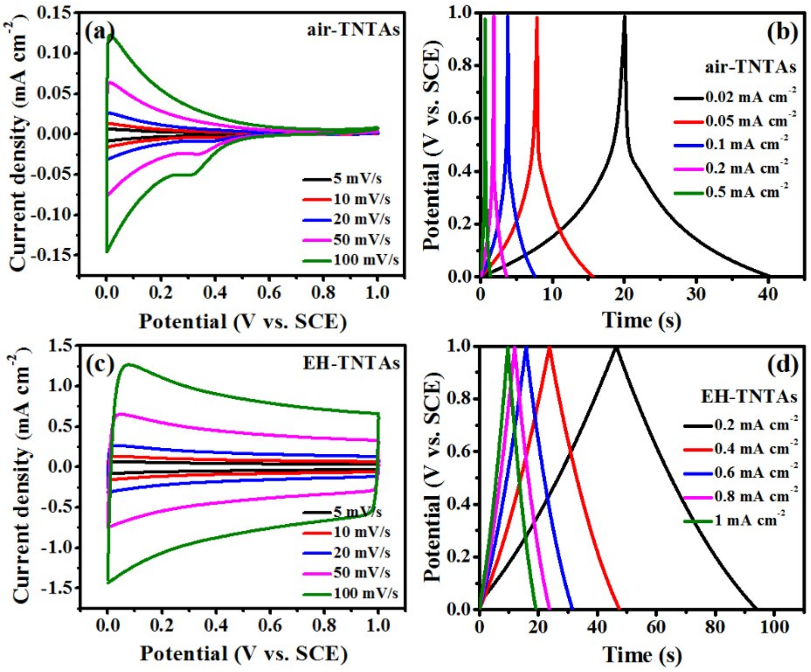

Fig. S2 CV curves of (a) air-TNTAs and (c) EH-TNTAs electrodes at different scan rates, GCD curves of (b) air-TNTAs and (d) EH-TNTAs electrodes at different current densities.

Table. S2 Areal capacitances of both air-TNTAs and EH-TNTAs at different scan rates

scan rates (mV cm^{-2})	air-TNTAs (mF cm^{-2})	EH-TNTAs (mF cm^{-2})
5	0.35	9.20
10	0.34	9.08
20	0.33	8.98
50	0.31	8.78
100	0.29	8.58

Table. S3 Areal capacitances of both air-TNTAs and EH-TNTAs at different current densities

current density (mA cm^{-2})	air-TNTAs (mF cm^{-2})	current density (mA cm^{-2})	EH-TNTAs (mF cm^{-2})
0.02	0.41	0.2	9.54
0.05	0.39	0.4	9.56
0.1	0.38	0.6	9.48
0.2	0.35	0.8	9.44
0.5	0.38	1.0	9.50

Table. S4 Fitting results of two different TNTAs electrodes based on the equivalent circuit

	R_s (ohm)	CPE (mF cm^{-2})	R_{ct} (ohm)	W (ohm)
air-TNTAs	7.674	0.00014	107.48	0.00091
EH-TNTAs	5.769	0.00112	16.03	0.00501

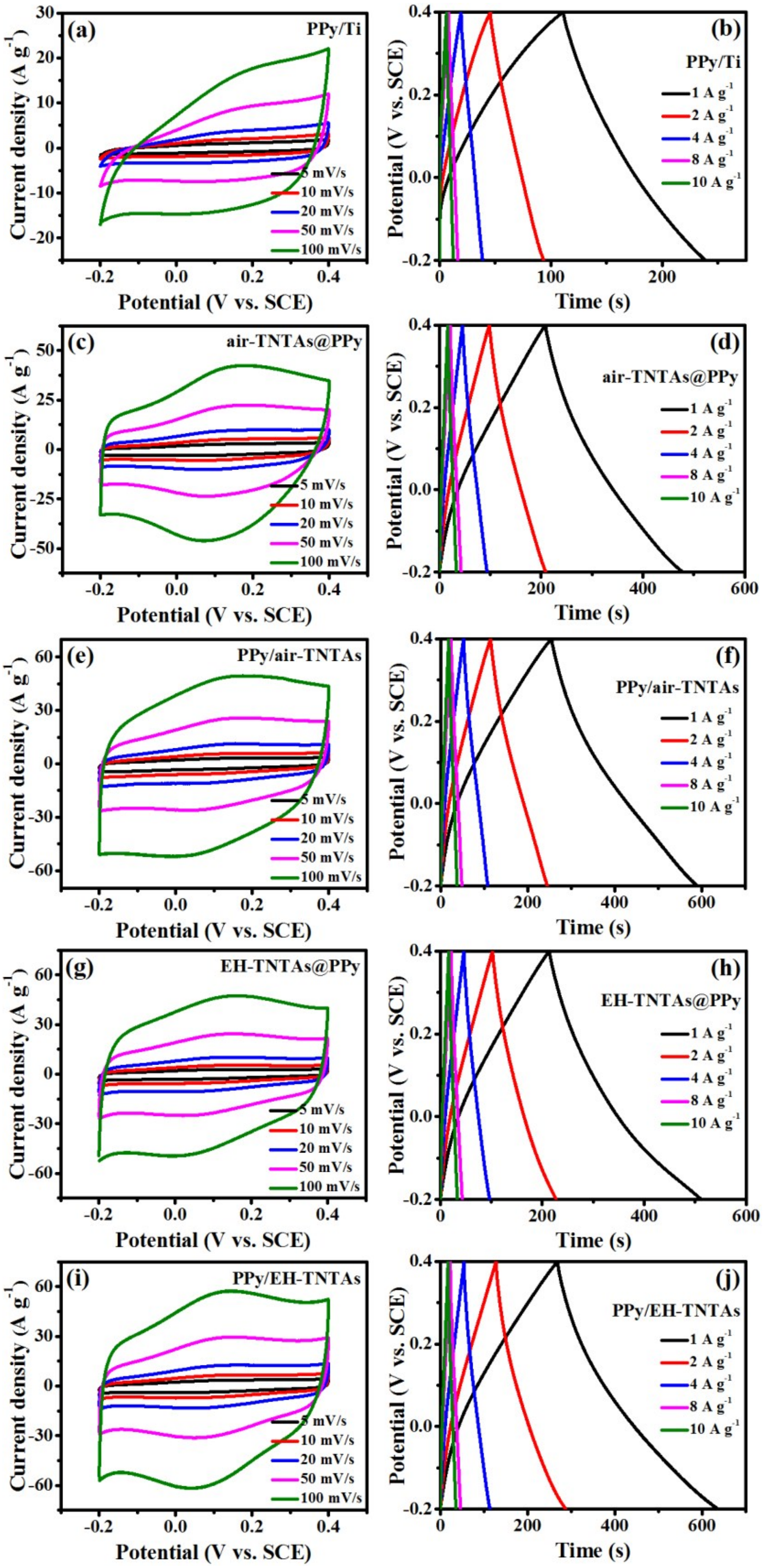

Fig. S3 CV curves of (a) PPy/Ti, (c) air-TNTAs@PPy, (e) PPy/air-TNTAs, (g) EH-TNTAs@PPy and (i) PPy/EH-TNTAs electrodes at different scan rates, GCD curves of (b) PPy/Ti, (d) air-TNTAs@PPy, (f) PPy/air-TNTAs, (h) EH-TNTAs@PPy and (j) PPy/EH-TNTAs electrodes at different current densities.

Table. S5 Specific capacitances of PPy/Ti and various PPy-TNTAs hybrid electrodes at different scan rates (based on the mass of PPy)

scan rates (mV s^{-1})	5	10	20	50	100
air-TNTAs@PPy (F g^{-1})	459.2	419.2	384.8	342.9	320.1
EH-TNTAs@PPy (F g^{-1})	465.2	435.6	414.2	387.4	371.8
PPy/air-TNTAs (F g^{-1})	515.0	474.1	444.0	408.4	391.1
PPy/EH-TNTAs (F g^{-1})	579.0	539.2	518.1	477.2	458.3
PPy/Ti (F g^{-1})	166.0	148.7	135.0	119.4	110.9

Table. S6 Specific capacitances of PPy/Ti and various PPy-TNTAs hybrid electrodes at different current densities (based on the mass of PPy)

current density (A g^{-1})	air-TNTAs@PPy(F g^{-1})	EH-TNTAs@PPy(F g^{-1})	PPy/air-TNTAs (F g^{-1})	PPy/EH-TNTAs(F g^{-1})	PPy/Ti (F g^{-1})
1	450.6	497.8	558.8	614.7	212.7
2	369.3	416.0	475.0	522.3	159.3
4	320.7	336.7	390.7	425.3	129.3
8	288.0	292.0	346.7	364.7	109.3
10	278.3	281.7	315.0	340.0	101.7

Table. S7 Fitting results of various PPy-containing hybrid electrodes based on the equivalent circuit

	R_s (ohm)	CPE_1 (mF cm^{-2})	R_{ct} (ohm)	CPE_2 (mF cm^{-2})
air-TNTAs@PPy	2.296	0.02086	9.152	0.00092
EH-TNTAs@PPy	1.519	0.02249	7.590	0.00169
PPy/air-TNTAs	0.872	0.03135	4.276	0.00102
PPy/EH-TNTAs	0.786	0.03495	3.528	0.00176

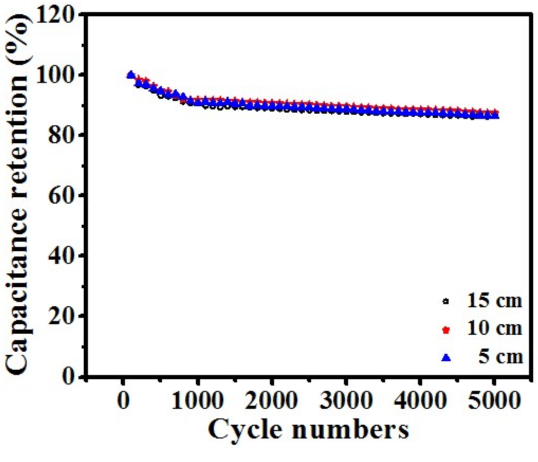

Fig.S4 Comparison of cycling stability of PPy/EH-TNTAs hybrid fabricated with different light intensities (the samples were respectively placed 5, 10 and 15 cm away from the light source with the

surface perpendicular to the light beam)

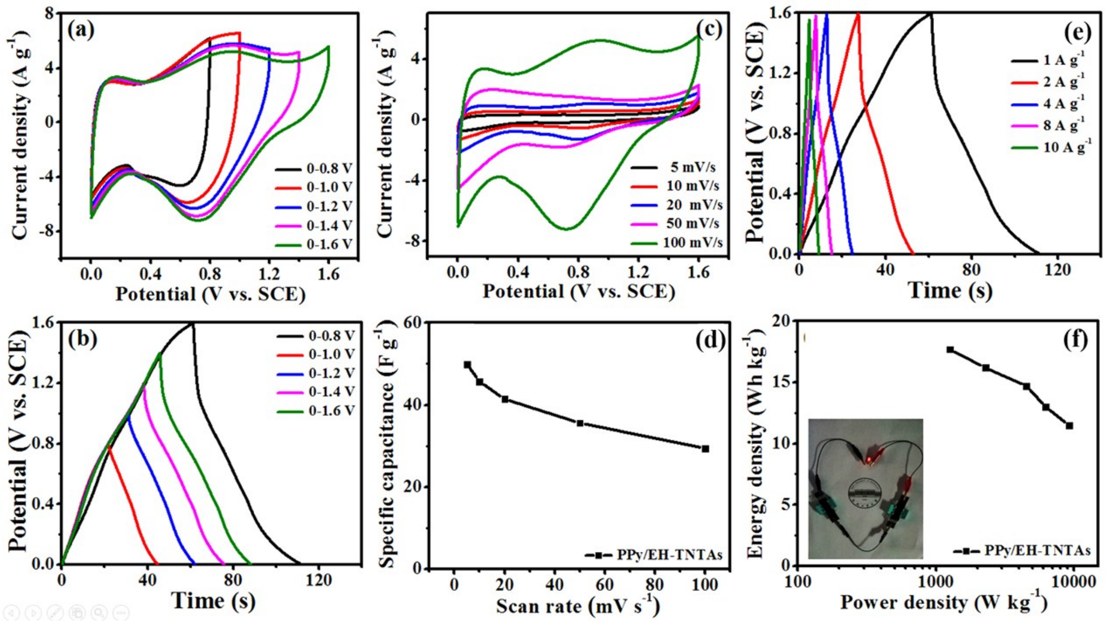

Fig.S5 Energy storage performance of the assembled symmetric supercapacitor device with PPy/EH-TNTAs as both positive and negative electrodes: CV(a) and GCD(b) curves with different working potential windows, CV curves(c) at different scan rates and its corresponding specific capacitances(d), GCD curves(e) and the corresponding Ragone plot(f)
